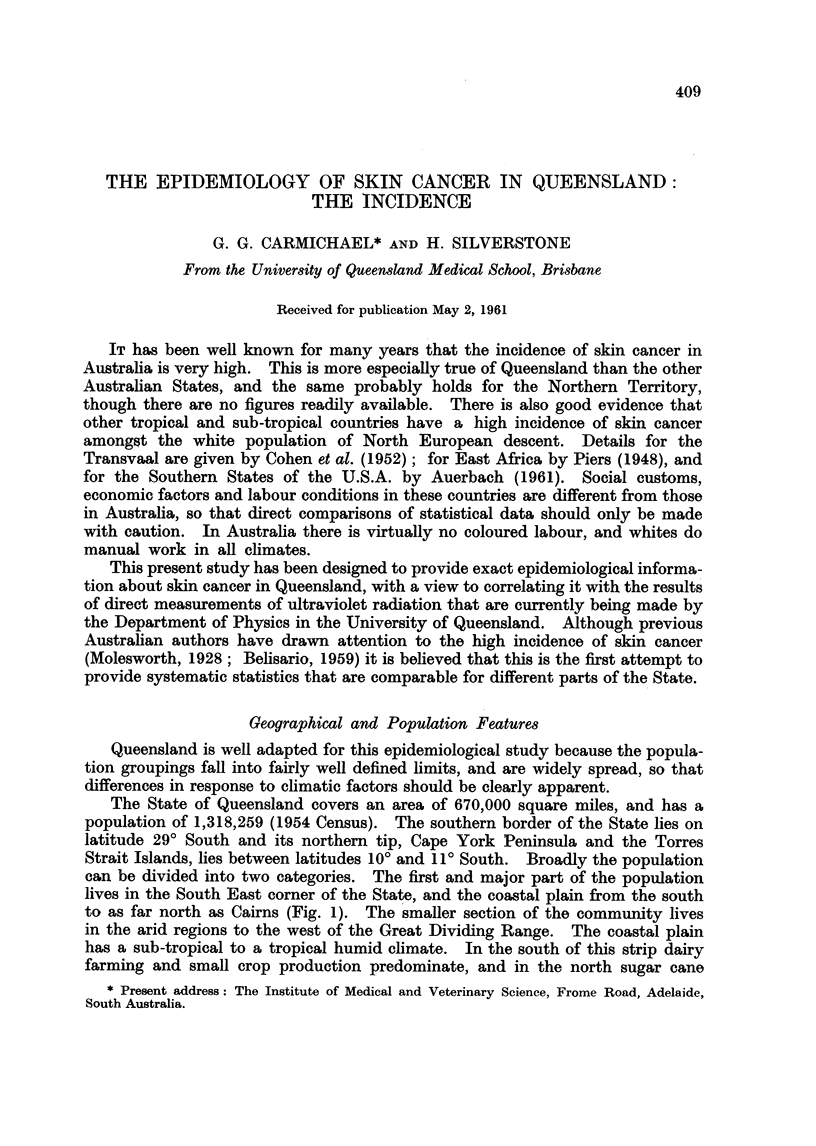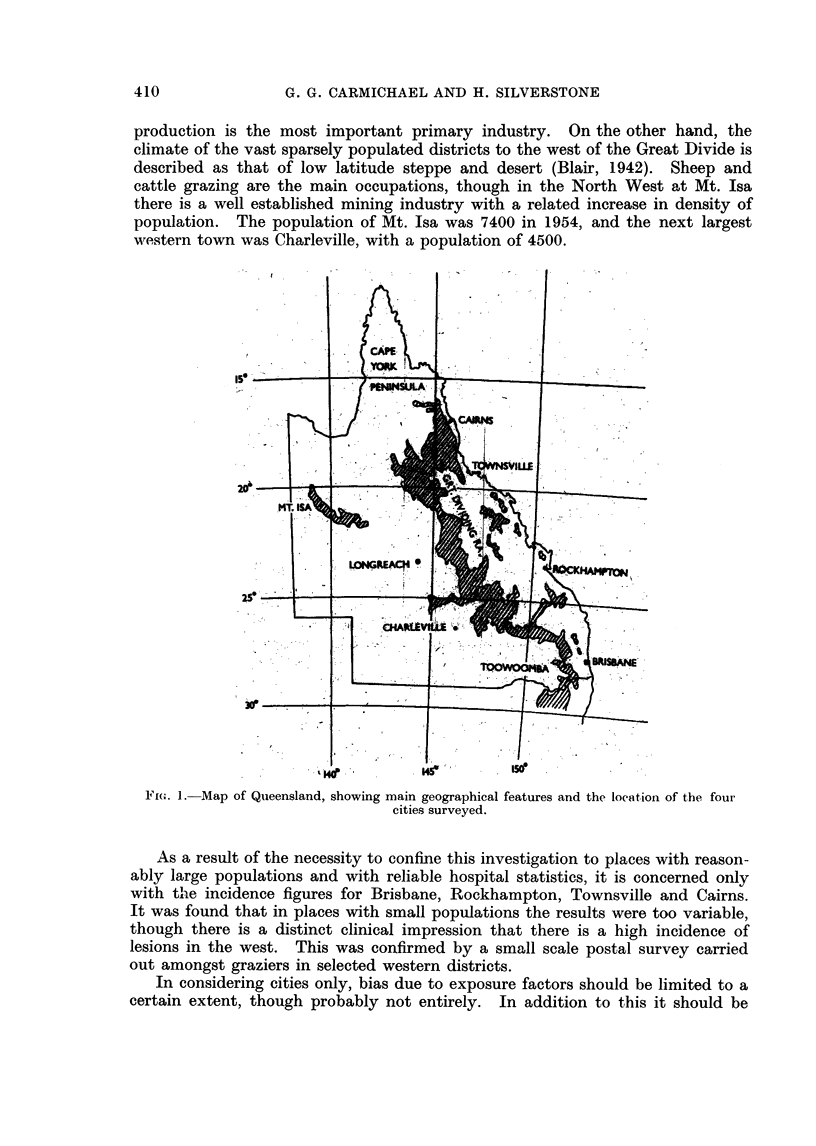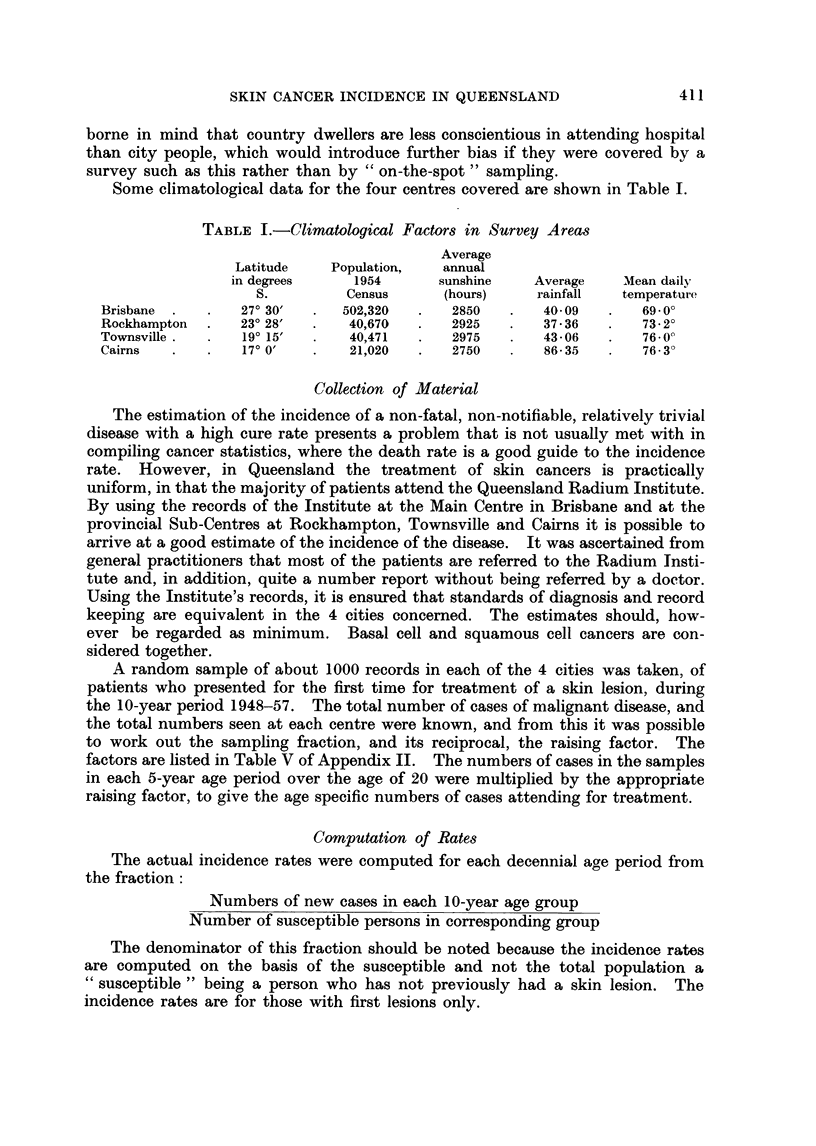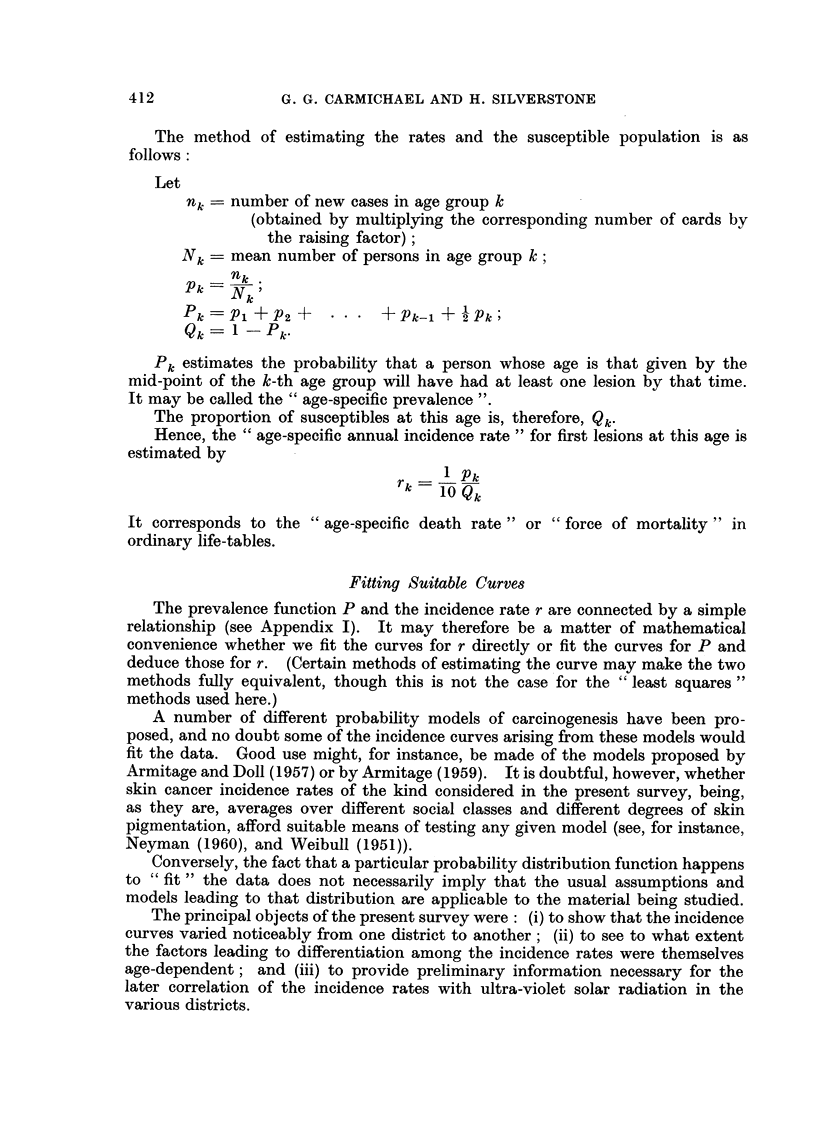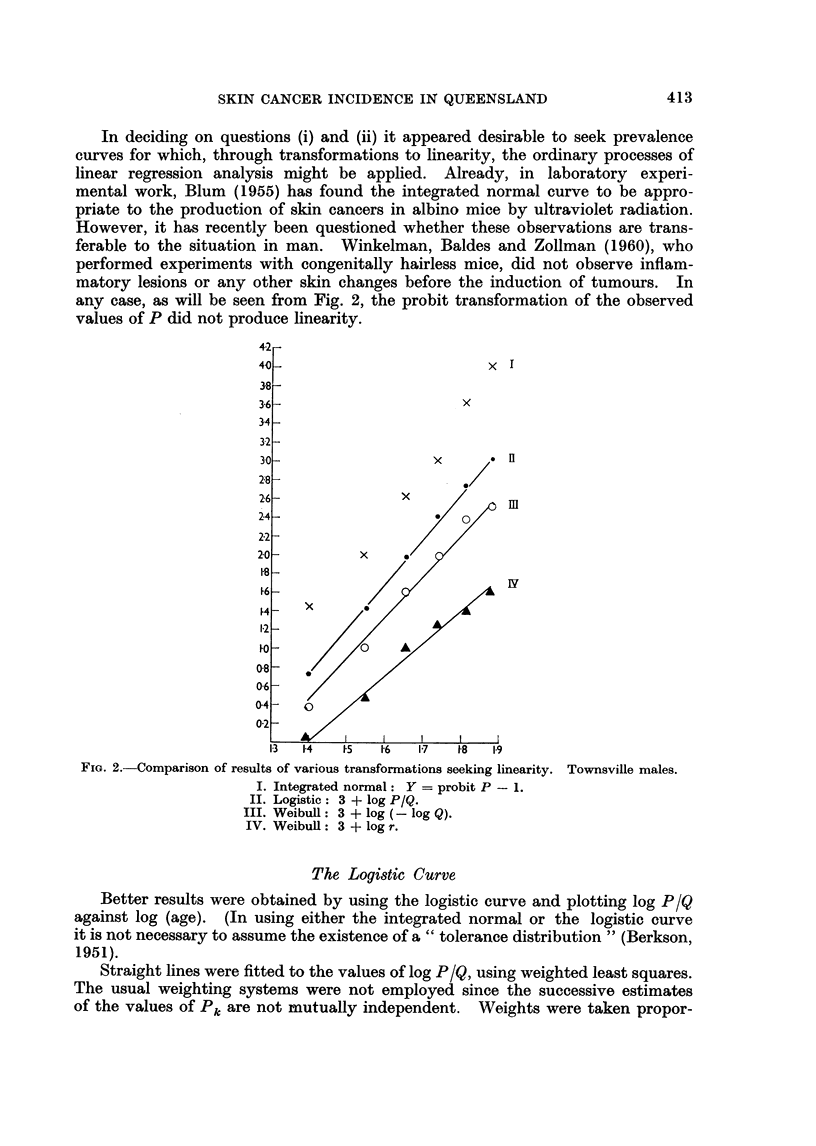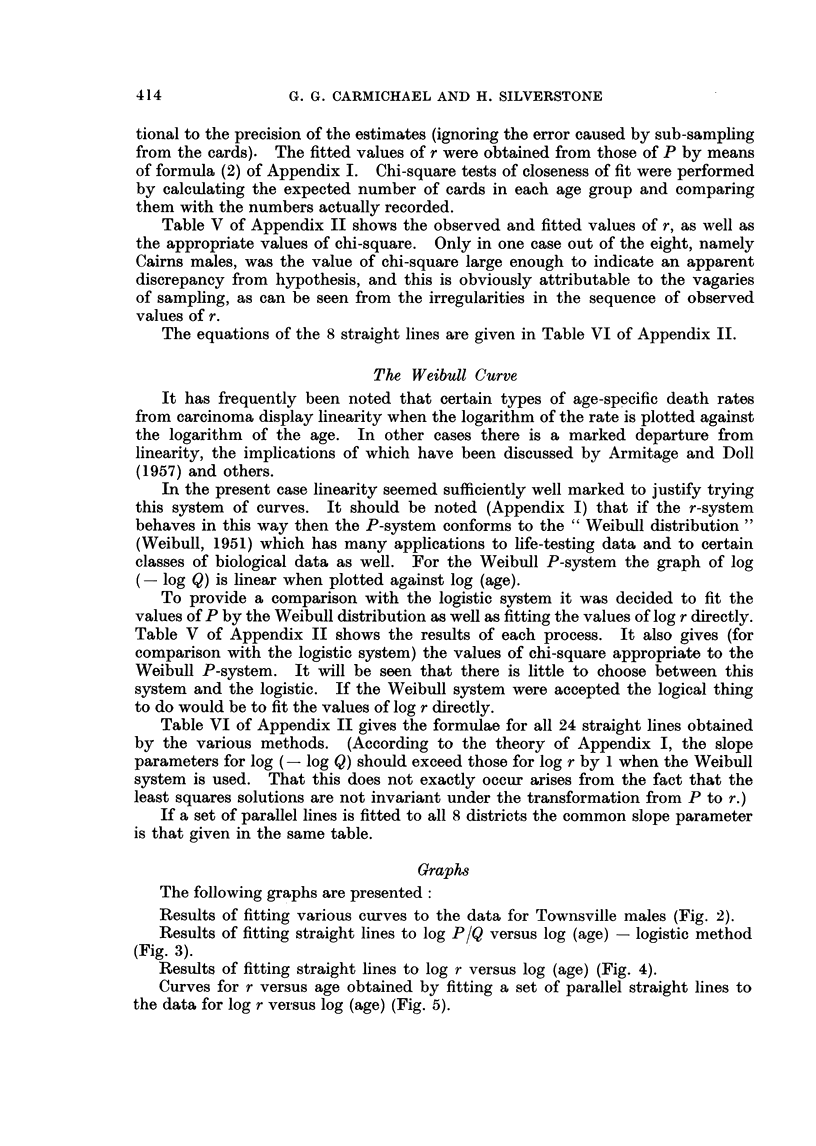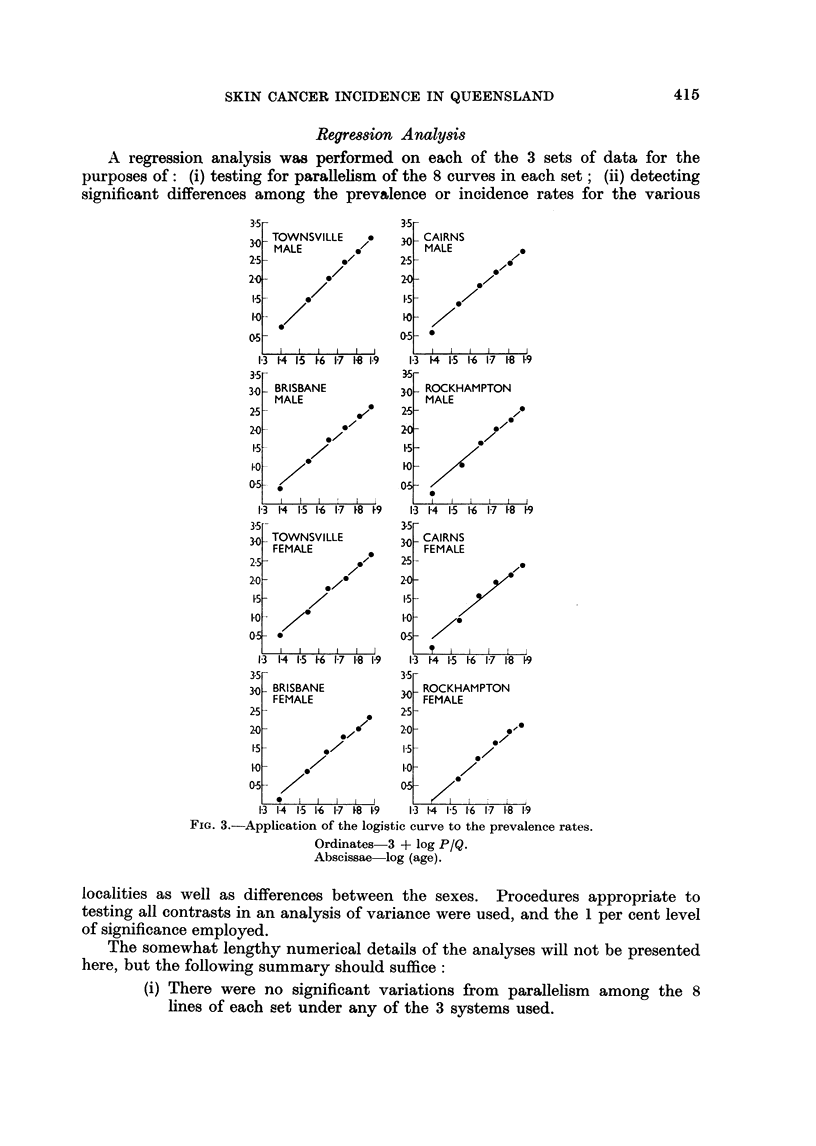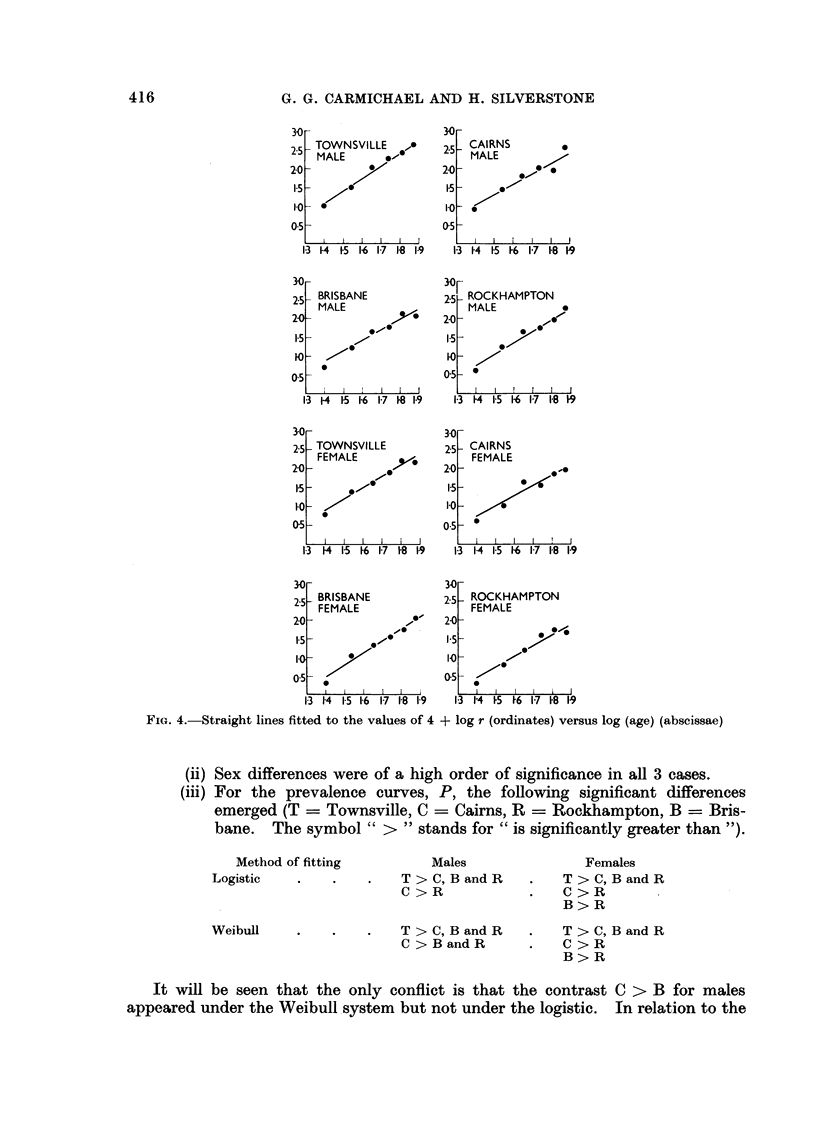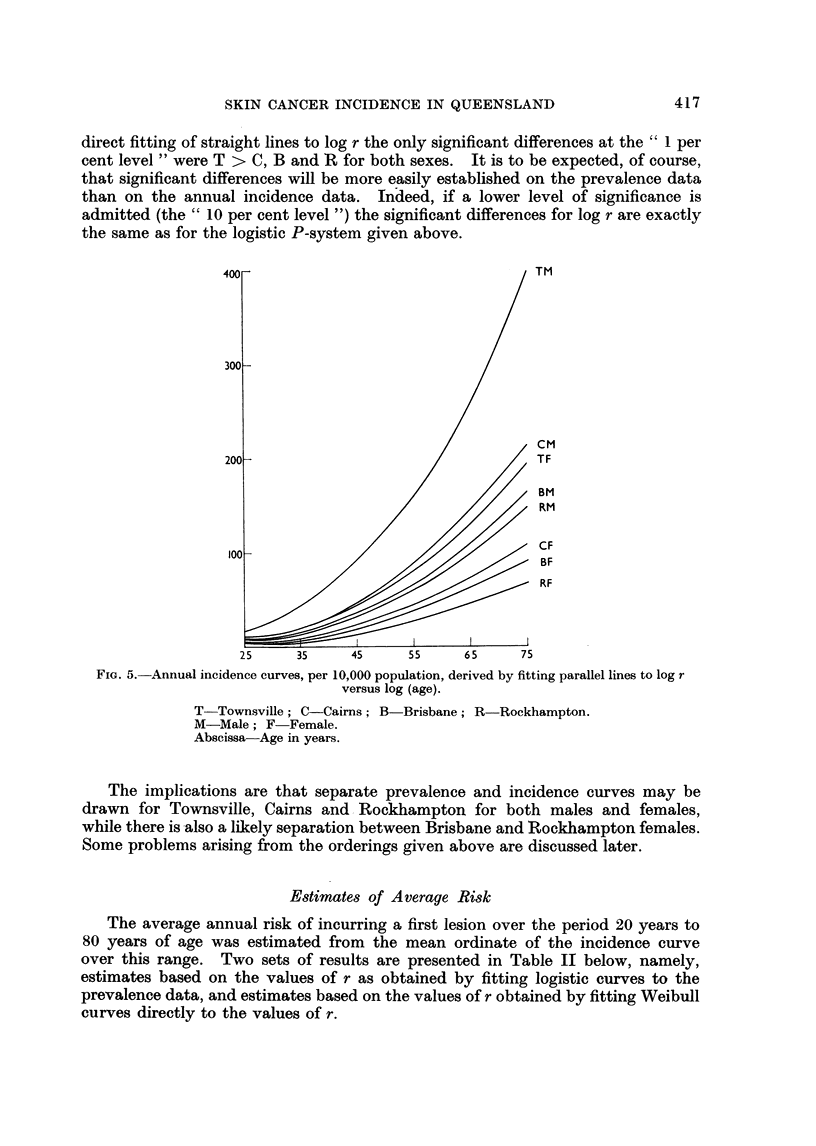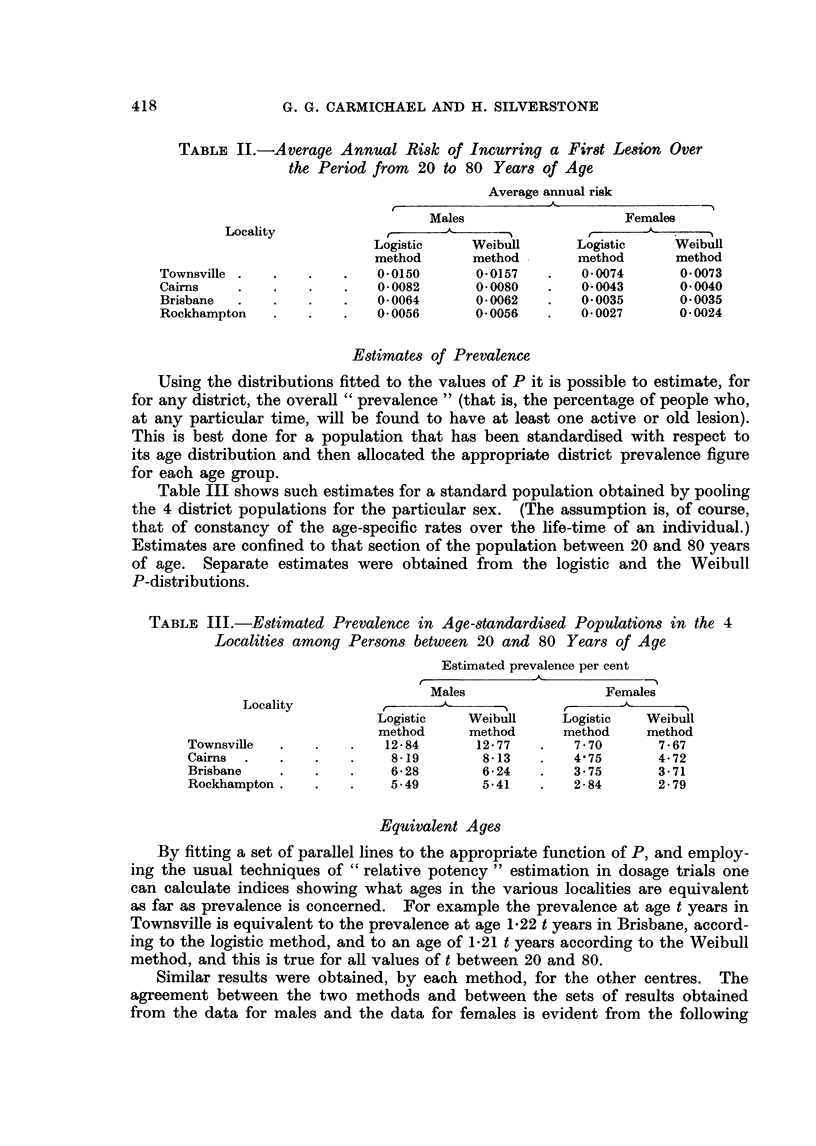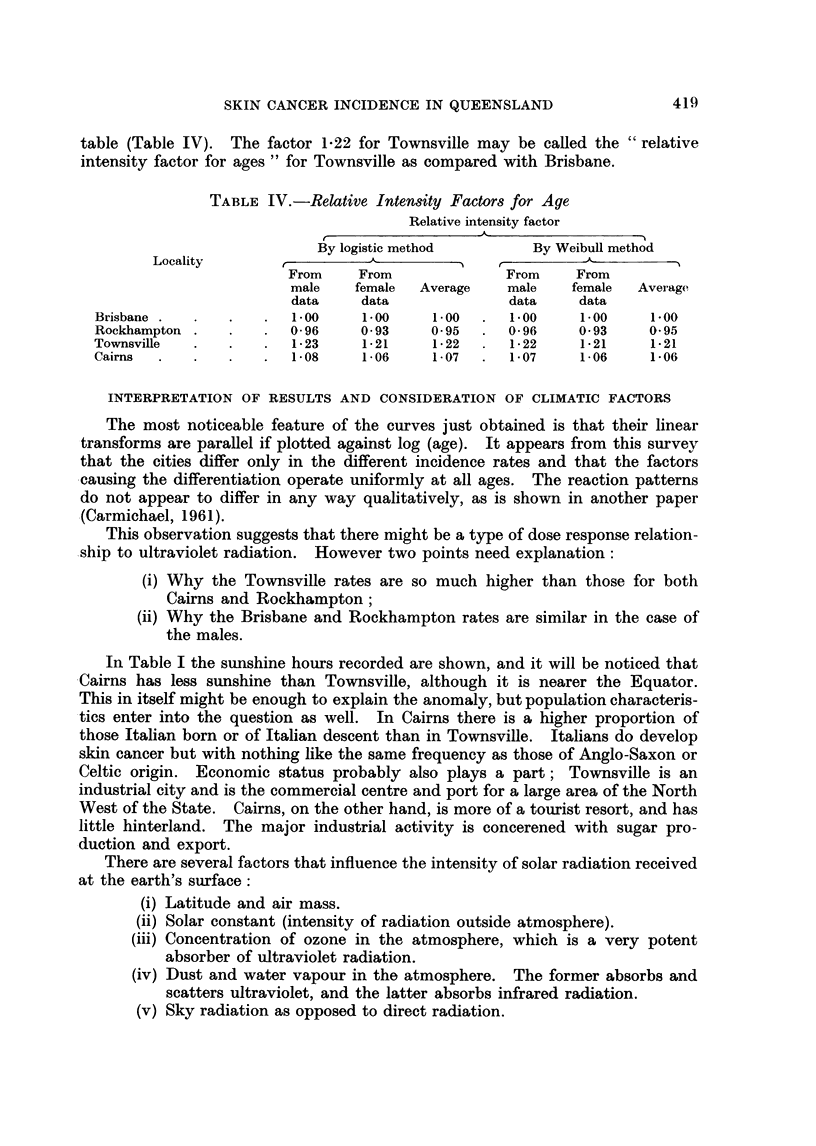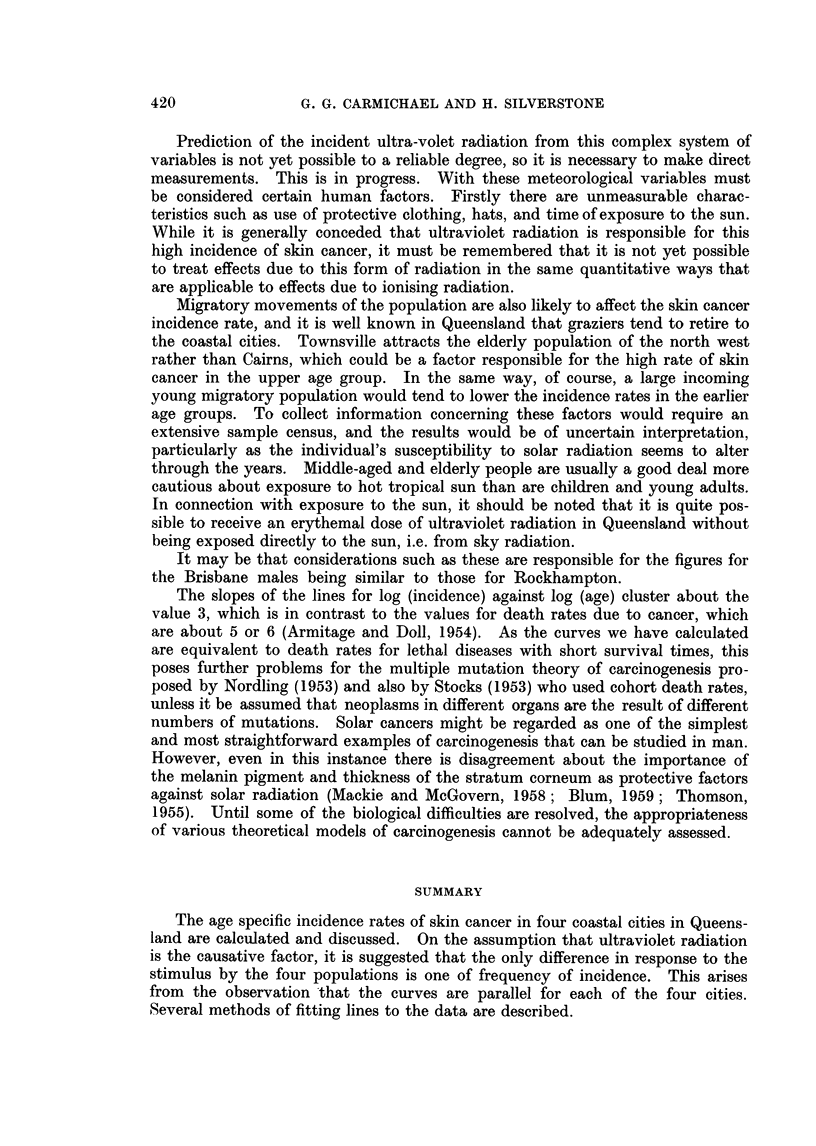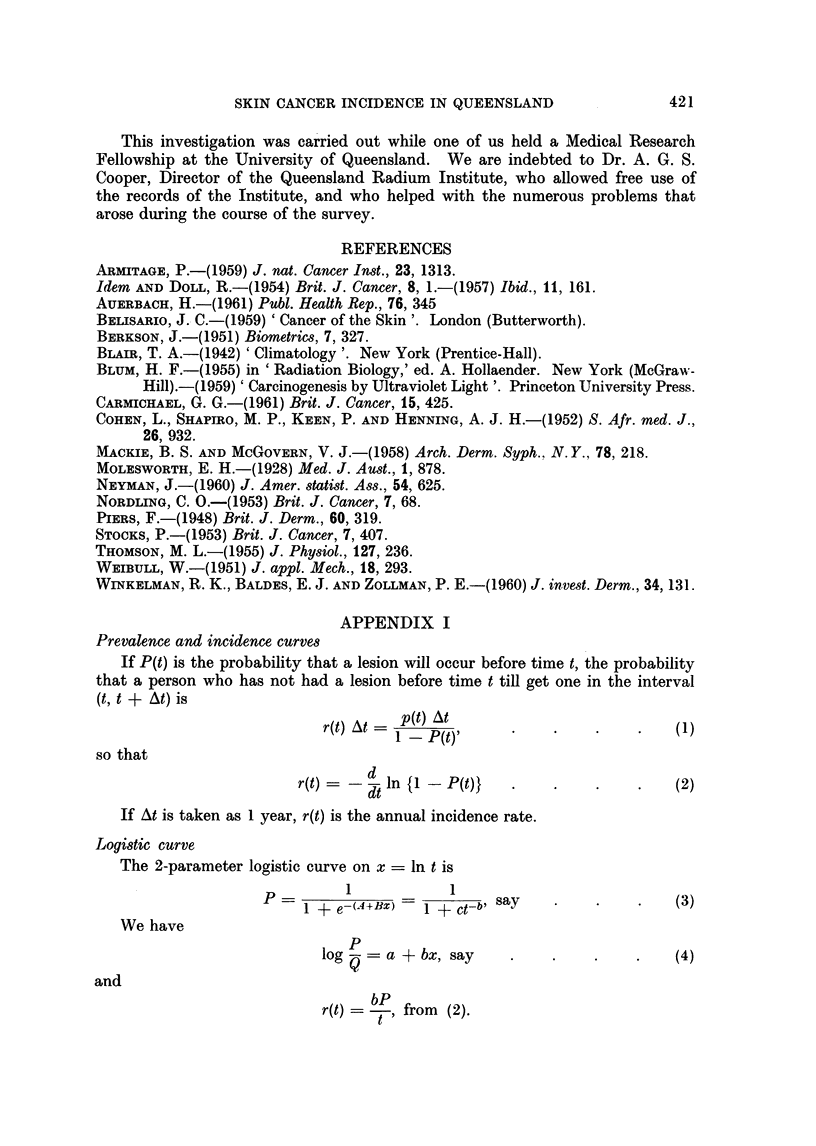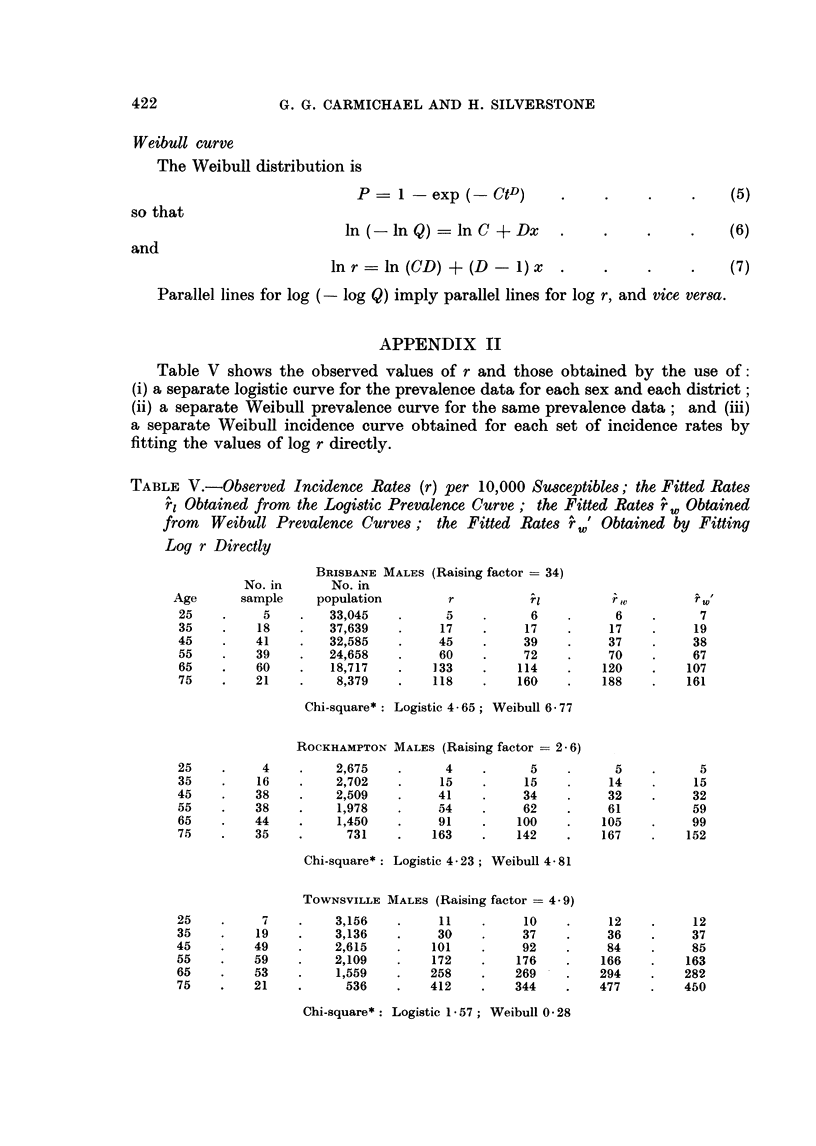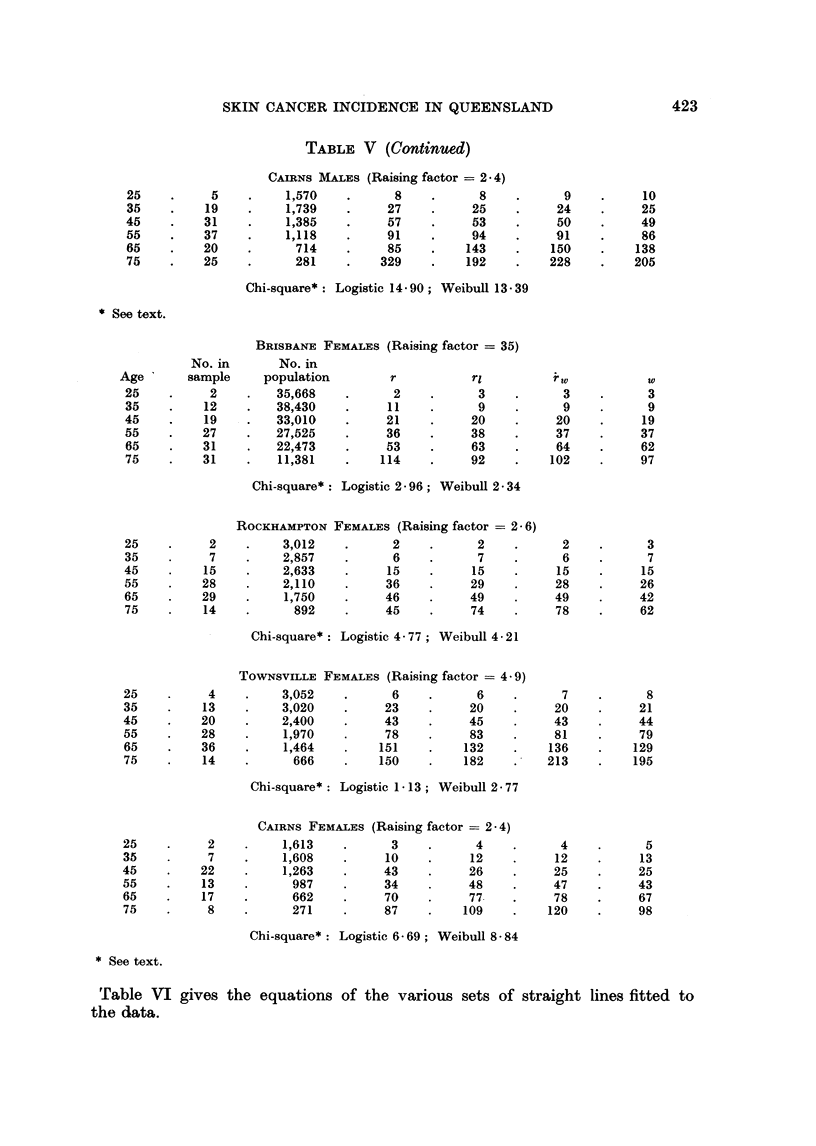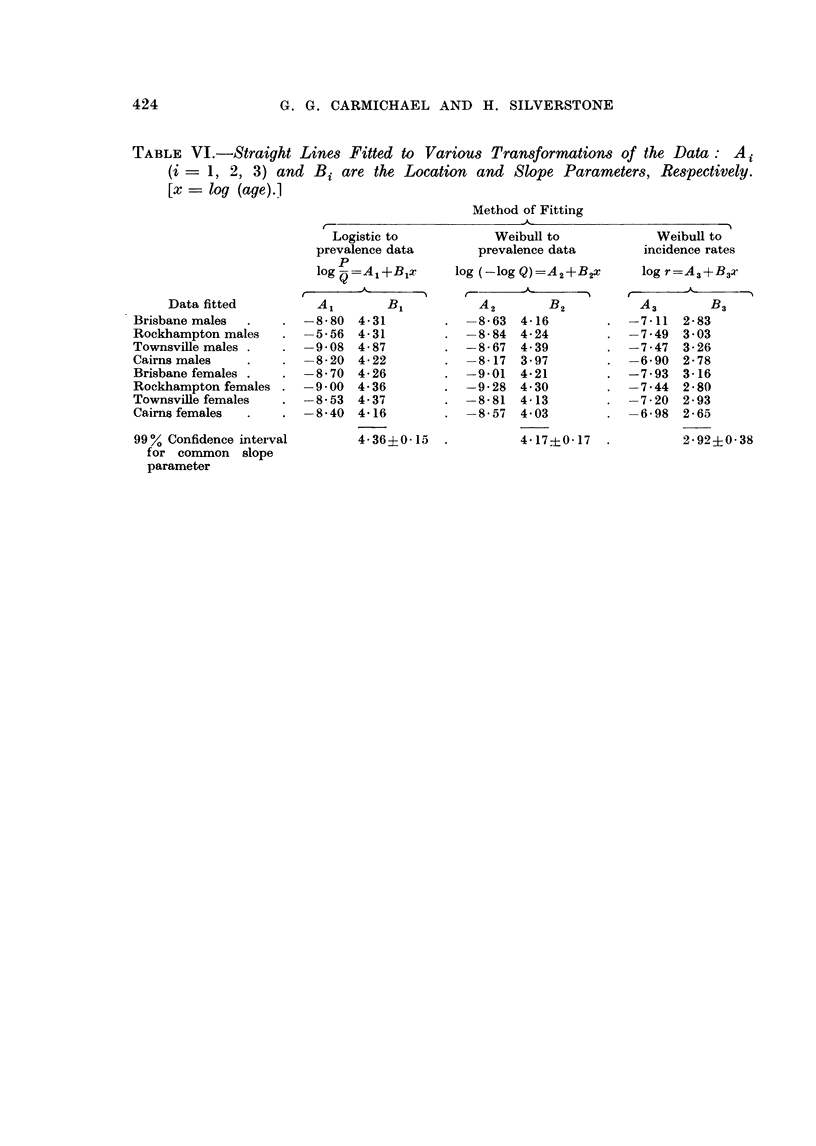# The Epidemiology of Skin Cancer in Queensland: The Incidence

**DOI:** 10.1038/bjc.1961.51

**Published:** 1961-09

**Authors:** G. G. Carmichael, H. Silverstone


### THE EPIDEMIOLOGY OF SKIN CANCER IN QUEENSLAND: THE INCIDENCE

G. G. CARMICHAEL* AND H. SILVERSTONE

*From the University of Queensland Medical School, Brisbane*

Received for publication May 2, 1961

IT has been well known for many years that the incidence of skin cancer in Australia is very high. This is more especially true of Queensland than the other Australian States, and the same probably holds for the Northern Territory, though there are no figures readily available. There is also good evidence that other tropical and sub-tropical countries have a high incidence of skin cancer amongst the white population of North European descent. Details for the Transvaal are given by Cohen *et al.* (1952); for East Africa by Piers (1948), and for the Southern States of the U.S.A. by Auerbach (1961). Social customs, economic factors and labour conditions in these countries are different from those in Australia, so that direct comparisons of statistical data should only be made with caution. In Australia there is virtually no coloured labour, and whites do manual work in all climates.

This present study has been designed to provide exact epidemiological information about skin cancer in Queensland, with a view to correlating it with the results of direct measurements of ultraviolet radiation that are currently being made by the Department of Physics in the University of Queensland. Although previous Australian authors have drawn attention to the high incidence of skin cancer (Molesworth, 1928; Belisario, 1959) it is believed that this is the first attempt to provide systematic statistics that are comparable for different parts of the State.

### *Geographical and Population Features*

Queensland is well adapted for this epidemiological study because the population groupings fall into fairly well defined limits, and are widely spread, so that differences in response to climatic factors should be clearly apparent.

The State of Queensland covers an area of 670,000 square miles, and has a population of 1,318,259 (1954 Census). The southern border of the State lies on latitude 29° South and its northern tip, Cape York Peninsula and the Torres Strait Islands, lies between latitudes 10° and 11° South. Broadly the population can be divided into two categories. The first and major part of the population lives in the South East corner of the State, and the coastal plain from the south to as far north as Cairns (Fig. 1). The smaller section of the community lives in the arid regions to the west of the Great Dividing Range. The coastal plain has a sub-tropical to a tropical humid climate. In the south of this strip dairy farming and small crop production predominate, and in the north sugar cane

* Present address: The Institute of Medical and Veterinary Science, Frome Road, Adelaide, South Australia.

production is the most important primary industry. On the other hand, the climate of the vast sparsely populated districts to the west of the Great Divide is described as that of low latitude steppe and desert (Blair, 1942). Sheep and cattle grazing are the main occupations, though in the North West at Mt. Isa there is a well established mining industry with a related increase in density of population. The population of Mt. Isa was 7400 in 1954, and the next largest western town was Charleville, with a population of 4500.

FIG. 1.—Map of Queensland, showing main geographical features and the location of the four cities surveyed.

As a result of the necessity to confine this investigation to places with reasonably large populations and with reliable hospital statistics, it is concerned only with the incidence figures for Brisbane, Rockhampton, Townsville and Cairns. It was found that in places with small populations the results were too variable, though there is a distinct clinical impression that there is a high incidence of lesions in the west. This was confirmed by a small scale postal survey carried out amongst graziers in selected western districts.

In considering cities only, bias due to exposure factors should be limited to a certain extent, though probably not entirely. In addition to this it should be borne in mind that country dwellers are less conscientious in attending hospital than city people, which would introduce further bias if they were covered by a survey such as this rather than by " on-the-spot " sampling.

Some climatological data for the four centres covered are shown in Table I.

TABLE I.—*Climatological Factors in Survey Areas*

| | Latitude in degrees S. | Population, 1954 Census | Average annual sunshine (hours) | Average rainfall | Mean daily temperature |
|---|---|---|---|---|---|
| Brisbane | 27° 30′ | 502,320 | 2850 | 40·09 | 69·0° |
| Rockhampton | 23° 28′ | 40,670 | 2925 | 37·36 | 73·2° |
| Townsville | 19° 15′ | 40,471 | 2975 | 43·06 | 76·0° |
| Cairns | 17° 0′ | 21,020 | 2750 | 86·35 | 76·3° |

*Collection of Material*

The estimation of the incidence of a non-fatal, non-notifiable, relatively trivial disease with a high cure rate presents a problem that is not usually met with in compiling cancer statistics, where the death rate is a good guide to the incidence rate. However, in Queensland the treatment of skin cancers is practically uniform, in that the majority of patients attend the Queensland Radium Institute. By using the records of the Institute at the Main Centre in Brisbane and at the provincial Sub-Centres at Rockhampton, Townsville and Cairns it is possible to arrive at a good estimate of the incidence of the disease. It was ascertained from general practitioners that most of the patients are referred to the Radium Institute and, in addition, quite a number report without being referred by a doctor. Using the Institute's records, it is ensured that standards of diagnosis and record keeping are equivalent in the 4 cities concerned. The estimates should, however be regarded as minimum. Basal cell and squamous cell cancers are considered together.

A random sample of about 1000 records in each of the 4 cities was taken, of patients who presented for the first time for treatment of a skin lesion, during the 10-year period 1948–57. The total number of cases of malignant disease, and the total numbers seen at each centre were known, and from this it was possible to work out the sampling fraction, and its reciprocal, the raising factor. The factors are listed in Table V of Appendix II. The numbers of cases in the samples in each 5-year age period over the age of 20 were multiplied by the appropriate raising factor, to give the age specific numbers of cases attending for treatment.

*Computation of Rates*

The actual incidence rates were computed for each decennial age period from the fraction :

$$\frac{\text{Numbers of new cases in each 10-year age group}}{\text{Number of susceptible persons in corresponding group}}$$

The denominator of this fraction should be noted because the incidence rates are computed on the basis of the susceptible and not the total population a " susceptible " being a person who has not previously had a skin lesion. The incidence rates are for those with first lesions only.

The method of estimating the rates and the susceptible population is as follows :

Let

$n_k$ = number of new cases in age group $k$
(obtained by multiplying the corresponding number of cards by the raising factor) ;

$N_k$ = mean number of persons in age group $k$ ;

$p_k = \frac{n_k}{N_k}$ ;

$P_k = p_1 + p_2 + \quad . \; . \; . \quad + p_{k-1} + \frac{1}{2} p_k$ ;

$Q_k = 1 - P_k$.

$P_k$ estimates the probability that a person whose age is that given by the mid-point of the $k$-th age group will have had at least one lesion by that time. It may be called the " age-specific prevalence ".

The proportion of susceptibles at this age is, therefore, $Q_k$.

Hence, the " age-specific annual incidence rate " for first lesions at this age is estimated by

$$r_k = \frac{1}{10} \frac{p_k}{Q_k}$$

It corresponds to the " age-specific death rate " or " force of mortality " in ordinary life-tables.

*Fitting Suitable Curves*

The prevalence function $P$ and the incidence rate $r$ are connected by a simple relationship (see Appendix I). It may therefore be a matter of mathematical convenience whether we fit the curves for $r$ directly or fit the curves for $P$ and deduce those for $r$. (Certain methods of estimating the curve may make the two methods fully equivalent, though this is not the case for the " least squares " methods used here.)

A number of different probability models of carcinogenesis have been proposed, and no doubt some of the incidence curves arising from these models would fit the data. Good use might, for instance, be made of the models proposed by Armitage and Doll (1957) or by Armitage (1959). It is doubtful, however, whether skin cancer incidence rates of the kind considered in the present survey, being, as they are, averages over different social classes and different degrees of skin pigmentation, afford suitable means of testing any given model (see, for instance, Neyman (1960), and Weibull (1951)).

Conversely, the fact that a particular probability distribution function happens to " fit " the data does not necessarily imply that the usual assumptions and models leading to that distribution are applicable to the material being studied.

The principal objects of the present survey were : (i) to show that the incidence curves varied noticeably from one district to another ; (ii) to see to what extent the factors leading to differentiation among the incidence rates were themselves age-dependent ; and (iii) to provide preliminary information necessary for the later correlation of the incidence rates with ultra-violet solar radiation in the various districts.

In deciding on questions (i) and (ii) it appeared desirable to seek prevalence curves for which, through transformations to linearity, the ordinary processes of linear regression analysis might be applied. Already, in laboratory experimental work, Blum (1955) has found the integrated normal curve to be appropriate to the production of skin cancers in albino mice by ultraviolet radiation. However, it has recently been questioned whether these observations are transferable to the situation in man. Winkelman, Baldes and Zollman (1960), who performed experiments with congenitally hairless mice, did not observe inflammatory lesions or any other skin changes before the induction of tumours. In any case, as will be seen from Fig. 2, the probit transformation of the observed values of $P$ did not produce linearity.

FIG. 2.—Comparison of results of various transformations seeking linearity. Townsville males.

I. Integrated normal: $Y =$ probit $P - 1$.

II. Logistic: $3 + \log P/Q$.

III. Weibull: $3 + \log(-\log Q)$.

IV. Weibull: $3 + \log r$.

### *The Logistic Curve*

Better results were obtained by using the logistic curve and plotting log $P/Q$ against log (age). (In using either the integrated normal or the logistic curve it is not necessary to assume the existence of a " tolerance distribution " (Berkson, 1951).

Straight lines were fitted to the values of log $P/Q$, using weighted least squares. The usual weighting systems were not employed since the successive estimates of the values of $P_k$ are not mutually independent. Weights were taken proportional to the precision of the estimates (ignoring the error caused by sub-sampling from the cards). The fitted values of $r$ were obtained from those of $P$ by means of formula (2) of Appendix I. Chi-square tests of closeness of fit were performed by calculating the expected number of cards in each age group and comparing them with the numbers actually recorded.

Table V of Appendix II shows the observed and fitted values of $r$, as well as the appropriate values of chi-square. Only in one case out of the eight, namely Cairns males, was the value of chi-square large enough to indicate an apparent discrepancy from hypothesis, and this is obviously attributable to the vagaries of sampling, as can be seen from the irregularities in the sequence of observed values of $r$.

The equations of the 8 straight lines are given in Table VI of Appendix II.

*The Weibull Curve*

It has frequently been noted that certain types of age-specific death rates from carcinoma display linearity when the logarithm of the rate is plotted against the logarithm of the age. In other cases there is a marked departure from linearity, the implications of which have been discussed by Armitage and Doll (1957) and others.

In the present case linearity seemed sufficiently well marked to justify trying this system of curves. It should be noted (Appendix I) that if the $r$-system behaves in this way then the $P$-system conforms to the " Weibull distribution " (Weibull, 1951) which has many applications to life-testing data and to certain classes of biological data as well. For the Weibull $P$-system the graph of log $(-\log Q)$ is linear when plotted against log (age).

To provide a comparison with the logistic system it was decided to fit the values of $P$ by the Weibull distribution as well as fitting the values of log $r$ directly. Table V of Appendix II shows the results of each process. It also gives (for comparison with the logistic system) the values of chi-square appropriate to the Weibull $P$-system. It will be seen that there is little to choose between this system and the logistic. If the Weibull system were accepted the logical thing to do would be to fit the values of log $r$ directly.

Table VI of Appendix II gives the formulae for all 24 straight lines obtained by the various methods. (According to the theory of Appendix I, the slope parameters for log $(-\log Q)$ should exceed those for log $r$ by 1 when the Weibull system is used. That this does not exactly occur arises from the fact that the least squares solutions are not invariant under the transformation from $P$ to $r$.)

If a set of parallel lines is fitted to all 8 districts the common slope parameter is that given in the same table.

*Graphs*

The following graphs are presented :

Results of fitting various curves to the data for Townsville males (Fig. 2).

Results of fitting straight lines to log $P/Q$ versus log (age) — logistic method (Fig. 3).

Results of fitting straight lines to log $r$ versus log (age) (Fig. 4).

Curves for $r$ versus age obtained by fitting a set of parallel straight lines to the data for log $r$ versus log (age) (Fig. 5).

### *Regression Analysis*

A regression analysis was performed on each of the 3 sets of data for the purposes of : (i) testing for parallelism of the 8 curves in each set ; (ii) detecting significant differences among the prevalence or incidence rates for the various

FIG. 3.—Application of the logistic curve to the prevalence rates.
Ordinates—3 + log $P/Q$.
Abscissae—log (age).

localities as well as differences between the sexes. Procedures appropriate to testing all contrasts in an analysis of variance were used, and the 1 per cent level of significance employed.

The somewhat lengthy numerical details of the analyses will not be presented here, but the following summary should suffice :

(i) There were no significant variations from parallelism among the 8 lines of each set under any of the 3 systems used.

Fig. 4.—Straight lines fitted to the values of 4 + log $r$ (ordinates) versus log (age) (abscissae)

(ii) Sex differences were of a high order of significance in all 3 cases.

(iii) For the prevalence curves, $P$, the following significant differences emerged (T = Townsville, C = Cairns, R = Rockhampton, B = Brisbane. The symbol " > " stands for " is significantly greater than ").

| Method of fitting | Males | Females |
|---|---|---|
| Logistic . . . | T > C, B and R . | T > C, B and R |
| | C > R . | C > R |
| | | B > R |
| Weibull . . . | T > C, B and R . | T > C, B and R |
| | C > B and R . | C > R |
| | | B > R |

It will be seen that the only conflict is that the contrast C > B for males appeared under the Weibull system but not under the logistic. In relation to the direct fitting of straight lines to log $r$ the only significant differences at the " 1 per cent level " were T $>$ C, B and R for both sexes. It is to be expected, of course, that significant differences will be more easily established on the prevalence data than on the annual incidence data. Indeed, if a lower level of significance is admitted (the " 10 per cent level ") the significant differences for log $r$ are exactly the same as for the logistic $P$-system given above.

FIG. 5.—Annual incidence curves, per 10,000 population, derived by fitting parallel lines to log $r$ versus log (age).

T—Townsville ; C—Cairns ; B—Brisbane ; R—Rockhampton.
M—Male ; F—Female.
Abscissa—Age in years.

The implications are that separate prevalence and incidence curves may be drawn for Townsville, Cairns and Rockhampton for both males and females, while there is also a likely separation between Brisbane and Rockhampton females. Some problems arising from the orderings given above are discussed later.

*Estimates of Average Risk*

The average annual risk of incurring a first lesion over the period 20 years to 80 years of age was estimated from the mean ordinate of the incidence curve over this range. Two sets of results are presented in Table II below, namely, estimates based on the values of $r$ as obtained by fitting logistic curves to the prevalence data, and estimates based on the values of $r$ obtained by fitting Weibull curves directly to the values of $r$.

TABLE II.—*Average Annual Risk of Incurring a First Lesion Over the Period from* 20 *to* 80 *Years of Age*

| Locality | Average annual risk: Males, Logistic method | Males, Weibull method | Females, Logistic method | Females, Weibull method |
|---|---|---|---|---|
| Townsville | 0·0150 | 0·0157 | 0·0074 | 0·0073 |
| Cairns | 0·0082 | 0·0080 | 0·0043 | 0·0040 |
| Brisbane | 0·0064 | 0·0062 | 0·0035 | 0·0035 |
| Rockhampton | 0·0056 | 0·0056 | 0·0027 | 0·0024 |

### *Estimates of Prevalence*

Using the distributions fitted to the values of $P$ it is possible to estimate, for for any district, the overall " prevalence " (that is, the percentage of people who, at any particular time, will be found to have at least one active or old lesion). This is best done for a population that has been standardised with respect to its age distribution and then allocated the appropriate district prevalence figure for each age group.

Table III shows such estimates for a standard population obtained by pooling the 4 district populations for the particular sex. (The assumption is, of course, that of constancy of the age-specific rates over the life-time of an individual.) Estimates are confined to that section of the population between 20 and 80 years of age. Separate estimates were obtained from the logistic and the Weibull $P$-distributions.

TABLE III.—*Estimated Prevalence in Age-standardised Populations in the* 4 *Localities among Persons between* 20 *and* 80 *Years of Age*

| Locality | Estimated prevalence per cent: Males, Logistic method | Males, Weibull method | Females, Logistic method | Females, Weibull method |
|---|---|---|---|---|
| Townsville | 12·84 | 12·77 | 7·70 | 7·67 |
| Cairns | 8·19 | 8·13 | 4·75 | 4·72 |
| Brisbane | 6·28 | 6·24 | 3·75 | 3·71 |
| Rockhampton | 5·49 | 5·41 | 2·84 | 2·79 |

### *Equivalent Ages*

By fitting a set of parallel lines to the appropriate function of $P$, and employing the usual techniques of " relative potency " estimation in dosage trials one can calculate indices showing what ages in the various localities are equivalent as far as prevalence is concerned. For example the prevalence at age $t$ years in Townsville is equivalent to the prevalence at age 1·22 $t$ years in Brisbane, according to the logistic method, and to an age of 1·21 $t$ years according to the Weibull method, and this is true for all values of $t$ between 20 and 80.

Similar results were obtained, by each method, for the other centres. The agreement between the two methods and between the sets of results obtained from the data for males and the data for females is evident from the following table (Table IV). The factor 1·22 for Townsville may be called the "relative intensity factor for ages" for Townsville as compared with Brisbane.

TABLE IV.—*Relative Intensity Factors for Age*

| Locality | Relative intensity factor: By logistic method, From male data | From female data | Average | By Weibull method, From male data | From female data | Average |
|---|---|---|---|---|---|---|
| Brisbane | 1·00 | 1·00 | 1·00 | 1·00 | 1·00 | 1·00 |
| Rockhampton | 0·96 | 0·93 | 0·95 | 0·96 | 0·93 | 0·95 |
| Townsville | 1·23 | 1·21 | 1·22 | 1·22 | 1·21 | 1·21 |
| Cairns | 1·08 | 1·06 | 1·07 | 1·07 | 1·06 | 1·06 |

### INTERPRETATION OF RESULTS AND CONSIDERATION OF CLIMATIC FACTORS

The most noticeable feature of the curves just obtained is that their linear transforms are parallel if plotted against log (age). It appears from this survey that the cities differ only in the different incidence rates and that the factors causing the differentiation operate uniformly at all ages. The reaction patterns do not appear to differ in any way qualitatively, as is shown in another paper (Carmichael, 1961).

This observation suggests that there might be a type of dose response relationship to ultraviolet radiation. However two points need explanation :

(i) Why the Townsville rates are so much higher than those for both Cairns and Rockhampton ;
(ii) Why the Brisbane and Rockhampton rates are similar in the case of the males.

In Table I the sunshine hours recorded are shown, and it will be noticed that Cairns has less sunshine than Townsville, although it is nearer the Equator. This in itself might be enough to explain the anomaly, but population characteristics enter into the question as well. In Cairns there is a higher proportion of those Italian born or of Italian descent than in Townsville. Italians do develop skin cancer but with nothing like the same frequency as those of Anglo-Saxon or Celtic origin. Economic status probably also plays a part ; Townsville is an industrial city and is the commercial centre and port for a large area of the North West of the State. Cairns, on the other hand, is more of a tourist resort, and has little hinterland. The major industrial activity is concerened with sugar production and export.

There are several factors that influence the intensity of solar radiation received at the earth's surface :

(i) Latitude and air mass.
(ii) Solar constant (intensity of radiation outside atmosphere).
(iii) Concentration of ozone in the atmosphere, which is a very potent absorber of ultraviolet radiation.
(iv) Dust and water vapour in the atmosphere. The former absorbs and scatters ultraviolet, and the latter absorbs infrared radiation.
(v) Sky radiation as opposed to direct radiation.

Prediction of the incident ultra-volet radiation from this complex system of variables is not yet possible to a reliable degree, so it is necessary to make direct measurements. This is in progress. With these meteorological variables must be considered certain human factors. Firstly there are unmeasurable characteristics such as use of protective clothing, hats, and time of exposure to the sun. While it is generally conceded that ultraviolet radiation is responsible for this high incidence of skin cancer, it must be remembered that it is not yet possible to treat effects due to this form of radiation in the same quantitative ways that are applicable to effects due to ionising radiation.

Migratory movements of the population are also likely to affect the skin cancer incidence rate, and it is well known in Queensland that graziers tend to retire to the coastal cities. Townsville attracts the elderly population of the north west rather than Cairns, which could be a factor responsible for the high rate of skin cancer in the upper age group. In the same way, of course, a large incoming young migratory population would tend to lower the incidence rates in the earlier age groups. To collect information concerning these factors would require an extensive sample census, and the results would be of uncertain interpretation, particularly as the individual's susceptibility to solar radiation seems to alter through the years. Middle-aged and elderly people are usually a good deal more cautious about exposure to hot tropical sun than are children and young adults. In connection with exposure to the sun, it should be noted that it is quite possible to receive an erythemal dose of ultraviolet radiation in Queensland without being exposed directly to the sun, i.e. from sky radiation.

It may be that considerations such as these are responsible for the figures for the Brisbane males being similar to those for Rockhampton.

The slopes of the lines for log (incidence) against log (age) cluster about the value 3, which is in contrast to the values for death rates due to cancer, which are about 5 or 6 (Armitage and Doll, 1954). As the curves we have calculated are equivalent to death rates for lethal diseases with short survival times, this poses further problems for the multiple mutation theory of carcinogenesis proposed by Nordling (1953) and also by Stocks (1953) who used cohort death rates, unless it be assumed that neoplasms in different organs are the result of different numbers of mutations. Solar cancers might be regarded as one of the simplest and most straightforward examples of carcinogenesis that can be studied in man. However, even in this instance there is disagreement about the importance of the melanin pigment and thickness of the stratum corneum as protective factors against solar radiation (Mackie and McGovern, 1958; Blum, 1959; Thomson, 1955). Until some of the biological difficulties are resolved, the appropriateness of various theoretical models of carcinogenesis cannot be adequately assessed.

### SUMMARY

The age specific incidence rates of skin cancer in four coastal cities in Queensland are calculated and discussed. On the assumption that ultraviolet radiation is the causative factor, it is suggested that the only difference in response to the stimulus by the four populations is one of frequency of incidence. This arises from the observation that the curves are parallel for each of the four cities. Several methods of fitting lines to the data are described.

This investigation was carried out while one of us held a Medical Research Fellowship at the University of Queensland. We are indebted to Dr. A. G. S. Cooper, Director of the Queensland Radium Institute, who allowed free use of the records of the Institute, and who helped with the numerous problems that arose during the course of the survey.

### REFERENCES

Armitage, P.—(1959) *J. nat. Cancer Inst.*, **23**, 1313.
*Idem* and Doll, R.—(1954) *Brit. J. Cancer*, **8**, 1.—(1957) *Ibid.*, **11**, 161.
Auerbach, H.—(1961) *Publ. Health Rep.*, **76**, 345
Belisario, J. C.—(1959) ' Cancer of the Skin '. London (Butterworth).
Berkson, J.—(1951) *Biometrics*, **7**, 327.
Blair, T. A.—(1942) ' Climatology '. New York (Prentice-Hall).
Blum, H. F.—(1955) in ' Radiation Biology,' ed. A. Hollaender. New York (McGraw-Hill).—(1959) ' Carcinogenesis by Ultraviolet Light '. Princeton University Press.
Carmichael, G. G.—(1961) *Brit. J. Cancer*, **15**, 425.
Cohen, L., Shapiro, M. P., Keen, P. and Henning, A. J. H.—(1952) *S. Afr. med. J.*, **26**, 932.
Mackie, B. S. and McGovern, V. J.—(1958) *Arch. Derm. Syph., N.Y.*, **78**, 218.
Molesworth, E. H.—(1928) *Med. J. Aust.*, **1**, 878.
Neyman, J.—(1960) *J. Amer. statist. Ass.*, **54**, 625.
Nordling, C. O.—(1953) *Brit. J. Cancer*, **7**, 68.
Piers, F.—(1948) *Brit. J. Derm.*, **60**, 319.
Stocks, P.—(1953) *Brit. J. Cancer*, **7**, 407.
Thomson, M. L.—(1955) *J. Physiol.*, **127**, 236.
Weibull, W.—(1951) *J. appl. Mech.*, **18**, 293.
Winkelman, R. K., Baldes, E. J. and Zollman, P. E.—(1960) *J. invest. Derm.*, **34**, 131.

### APPENDIX I

*Prevalence and incidence curves*

If $P(t)$ is the probability that a lesion will occur before time $t$, the probability that a person who has not had a lesion before time $t$ till get one in the interval $(t, t + \Delta t)$ is

$$r(t)\ \Delta t = \frac{p(t)\ \Delta t}{1 - P(t)}, \qquad (1)$$

so that

$$r(t) = -\frac{d}{dt} \ln \{1 - P(t)\} \qquad (2)$$

If $\Delta t$ is taken as 1 year, $r(t)$ is the annual incidence rate.

*Logistic curve*

The 2-parameter logistic curve on $x = \ln t$ is

$$P = \frac{1}{1 + e^{-(A+Bx)}} = \frac{1}{1 + ct^{-b}}, \text{ say} \qquad (3)$$

We have

$$\log \frac{P}{Q} = a + bx, \text{ say} \qquad (4)$$

and

$$r(t) = \frac{bP}{t}, \text{ from (2).}$$

*Weibull curve*

The Weibull distribution is

$$P = 1 - \exp(-Ct^D) \quad . \quad . \quad . \quad . \quad (5)$$

so that

$$\ln(-\ln Q) = \ln C + Dx \quad . \quad . \quad . \quad . \quad (6)$$

and

$$\ln r = \ln(CD) + (D-1)x \quad . \quad . \quad . \quad . \quad (7)$$

Parallel lines for log (— log $Q$) imply parallel lines for log $r$, and *vice versa.*

### APPENDIX II

Table V shows the observed values of $r$ and those obtained by the use of: (i) a separate logistic curve for the prevalence data for each sex and each district; (ii) a separate Weibull prevalence curve for the same prevalence data; and (iii) a separate Weibull incidence curve obtained for each set of incidence rates by fitting the values of log $r$ directly.

TABLE V.—*Observed Incidence Rates* ($r$) *per* 10,000 *Susceptibles; the Fitted Rates* $\hat{r}_l$ *Obtained from the Logistic Prevalence Curve; the Fitted Rates* $\hat{r}_w$ *Obtained from Weibull Prevalence Curves; the Fitted Rates* $\hat{r}_w'$ *Obtained by Fitting Log* $r$ *Directly*

BRISBANE MALES (Raising factor = 34)

| Age | No. in sample | No. in population | $r$ | $\hat{r}_l$ | $\hat{r}_w$ | $\hat{r}_w'$ |
|---|---|---|---|---|---|---|
| 25 | 5 | 33,045 | 5 | 6 | 6 | 7 |
| 35 | 18 | 37,639 | 17 | 17 | 17 | 19 |
| 45 | 41 | 32,585 | 45 | 39 | 37 | 38 |
| 55 | 39 | 24,658 | 60 | 72 | 70 | 67 |
| 65 | 60 | 18,717 | 133 | 114 | 120 | 107 |
| 75 | 21 | 8,379 | 118 | 160 | 188 | 161 |

Chi-square*: Logistic 4·65; Weibull 6·77

ROCKHAMPTON MALES (Raising factor = 2·6)

| Age | No. in sample | No. in population | $r$ | $\hat{r}_l$ | $\hat{r}_w$ | $\hat{r}_w'$ |
|---|---|---|---|---|---|---|
| 25 | 4 | 2,675 | 4 | 5 | 5 | 5 |
| 35 | 16 | 2,702 | 15 | 15 | 14 | 15 |
| 45 | 38 | 2,509 | 41 | 34 | 32 | 32 |
| 55 | 38 | 1,978 | 54 | 62 | 61 | 59 |
| 65 | 44 | 1,450 | 91 | 100 | 105 | 99 |
| 75 | 35 | 731 | 163 | 142 | 167 | 152 |

Chi-square*: Logistic 4·23; Weibull 4·81

TOWNSVILLE MALES (Raising factor = 4·9)

| Age | No. in sample | No. in population | $r$ | $\hat{r}_l$ | $\hat{r}_w$ | $\hat{r}_w'$ |
|---|---|---|---|---|---|---|
| 25 | 7 | 3,156 | 11 | 10 | 12 | 12 |
| 35 | 19 | 3,136 | 30 | 37 | 36 | 37 |
| 45 | 49 | 2,615 | 101 | 92 | 84 | 85 |
| 55 | 59 | 2,109 | 172 | 176 | 166 | 163 |
| 65 | 53 | 1,559 | 258 | 269 | 294 | 282 |
| 75 | 21 | 536 | 412 | 344 | 477 | 450 |

Chi-square*: Logistic 1·57; Weibull 0·28

TABLE V (*Continued*)

CAIRNS MALES (Raising factor = 2·4)

| | | | | | | |
|---|---|---|---|---|---|---|
| 25 | 5 | 1,570 | 8 | 8 | 9 | 10 |
| 35 | 19 | 1,739 | 27 | 25 | 24 | 25 |
| 45 | 31 | 1,385 | 57 | 53 | 50 | 49 |
| 55 | 37 | 1,118 | 91 | 94 | 91 | 86 |
| 65 | 20 | 714 | 85 | 143 | 150 | 138 |
| 75 | 25 | 281 | 329 | 192 | 228 | 205 |

Chi-square* : Logistic 14·90 ; Weibull 13·39

* See text.

BRISBANE FEMALES (Raising factor = 35)

| Age | No. in sample | No. in population | $r$ | $r_l$ | $\hat{r}_w$ | $w$ |
|---|---|---|---|---|---|---|
| 25 | 2 | 35,668 | 2 | 3 | 3 | 3 |
| 35 | 12 | 38,430 | 11 | 9 | 9 | 9 |
| 45 | 19 | 33,010 | 21 | 20 | 20 | 19 |
| 55 | 27 | 27,525 | 36 | 38 | 37 | 37 |
| 65 | 31 | 22,473 | 53 | 63 | 64 | 62 |
| 75 | 31 | 11,381 | 114 | 92 | 102 | 97 |

Chi-square* : Logistic 2·96 ; Weibull 2·34

ROCKHAMPTON FEMALES (Raising factor = 2·6)

| | | | | | | |
|---|---|---|---|---|---|---|
| 25 | 2 | 3,012 | 2 | 2 | 2 | 3 |
| 35 | 7 | 2,857 | 6 | 7 | 6 | 7 |
| 45 | 15 | 2,633 | 15 | 15 | 15 | 15 |
| 55 | 28 | 2,110 | 36 | 29 | 28 | 26 |
| 65 | 29 | 1,750 | 46 | 49 | 49 | 42 |
| 75 | 14 | 892 | 45 | 74 | 78 | 62 |

Chi-square* : Logistic 4·77 ; Weibull 4·21

TOWNSVILLE FEMALES (Raising factor = 4·9)

| | | | | | | |
|---|---|---|---|---|---|---|
| 25 | 4 | 3,052 | 6 | 6 | 7 | 8 |
| 35 | 13 | 3,020 | 23 | 20 | 20 | 21 |
| 45 | 20 | 2,400 | 43 | 45 | 43 | 44 |
| 55 | 28 | 1,970 | 78 | 83 | 81 | 79 |
| 65 | 36 | 1,464 | 151 | 132 | 136 | 129 |
| 75 | 14 | 666 | 150 | 182 | 213 | 195 |

Chi-square* : Logistic 1·13 ; Weibull 2·77

CAIRNS FEMALES (Raising factor = 2·4)

| | | | | | | |
|---|---|---|---|---|---|---|
| 25 | 2 | 1,613 | 3 | 4 | 4 | 5 |
| 35 | 7 | 1,608 | 10 | 12 | 12 | 13 |
| 45 | 22 | 1,263 | 43 | 26 | 25 | 25 |
| 55 | 13 | 987 | 34 | 48 | 47 | 43 |
| 65 | 17 | 662 | 70 | 77 | 78 | 67 |
| 75 | 8 | 271 | 87 | 109 | 120 | 98 |

Chi-square* : Logistic 6·69 ; Weibull 8·84

* See text.

Table VI gives the equations of the various sets of straight lines fitted to the data.

TABLE VI.—*Straight Lines Fitted to Various Transformations of the Data : $A_i$ ($i$ = 1, 2, 3) and $B_i$ are the Location and Slope Parameters, Respectively.* [$x$ = *log (age)*.]

| | Method of Fitting | | | | | |
|---|---|---|---|---|---|---|
| | Logistic to prevalence data $\log \frac{P}{Q} = A_1 + B_1 x$ | | Weibull to prevalence data $\log(-\log Q) = A_2 + B_2 x$ | | Weibull to incidence rates $\log r = A_3 + B_3 x$ | |
| Data fitted | $A_1$ | $B_1$ | $A_2$ | $B_2$ | $A_3$ | $B_3$ |
| Brisbane males | −8·80 | 4·31 | −8·63 | 4·16 | −7·11 | 2·83 |
| Rockhampton males | −5·56 | 4·31 | −8·84 | 4·24 | −7·49 | 3·03 |
| Townsville males | −9·08 | 4·87 | −8·67 | 4·39 | −7·47 | 3·26 |
| Cairns males | −8·20 | 4·22 | −8·17 | 3·97 | −6·90 | 2·78 |
| Brisbane females | −8·70 | 4·26 | −9·01 | 4·21 | −7·93 | 3·16 |
| Rockhampton females | −9·00 | 4·36 | −9·28 | 4·30 | −7·44 | 2·80 |
| Townsville females | −8·53 | 4·37 | −8·81 | 4·13 | −7·20 | 2·93 |
| Cairns females | −8·40 | 4·16 | −8·57 | 4·03 | −6·98 | 2·65 |
| 99% Confidence interval for common slope parameter | | 4·36±0·15 | | 4·17±0·17 | | 2·92±0·38 |